# Anti-ischemic therapy and stress testing: pathophysiologic, diagnostic and prognostic implications

**DOI:** 10.1186/1476-7120-2-14

**Published:** 2004-08-20

**Authors:** Rosa Sicari

**Affiliations:** 1 CNR, Institute of Clinical Physiology, Pisa, Italy

## Abstract

Anti-ischemic therapy, in particular beta-blockers, is the most commonly employed drug for the control of myocardial ischemia in patients with stable coronary artery disease. Its widespread use also in patients with suspected coronary artery disease has important practical, clinical diagnostic and prognostic implications because diagnostic tests are heavily influenced by its effects. In the present review, the pathophysiological mechanisms of ischemia protection by antianginal therapy are described. Not all stressors are created equal in front of the different classes of antianginal drugs and on their turn the different classes of drugs exert different levels of protection on inducible ischemia. Several clinical implications can be drawn: From the diagnostic viewpoint antianginal therapy decreases test sensitivity, offsetting the real ischemic burden for a too high percentage of false negative tests. From the prognostic viewpoint test positivity in medical therapy identifies a group of subjects at higher risk of experiencing cardiac death and positivity on medical therapy can be considered a parameter of ischemia severity. Nonetheless in patients with known coronary artery disease the ability of antianginal therapy to modify the ischemic threshold at stress testing represent a powerful means to assess therapy efficacy. From a practical viewpoint, the use of antianginal therapy at time of testing has advantages and disadvantages which are largely dependent on the purpose a test is performed: if the purpose of testing is to diagnose ischemia, it should be performed in the absence of antianginal medications. If the purpose of testing is to assess the protective effects of antianginal therapy, the test should be performed on medications.

## Background

Anti-ischemic therapy, in particular beta-blockers, is the most commonly employed drug for the control of myocardial ischemia in patients with stable coronary artery disease. Its widespread use also in patients with suspected coronary artery disease has important practical, clinical diagnostic and prognostic implications because diagnostic tests are heavily influenced by its effects. The diagnostic and prognostic impact of anti-ischemic therapy on stress testing is largely ignored but not negligible. The issue raises several questions: How to evaluate patients at time of testing for myocardial ischemia? How to interpret a stress test performed on anti-ischemic therapy? Are the stressors employed for the detection of myocardial ischemia created equal in relation to the different classes of drugs used in clinical practice? Is stress testing able to assess the efficacy of medical therapy in patients with known coronary artery disease? Has the protection of anti-ischemic therapy on inducible myocardial ischemia any impact on long-term survival?

## Pathophysiologic implications of anti-ischemic therapy during stress testing

**Figure 1:**
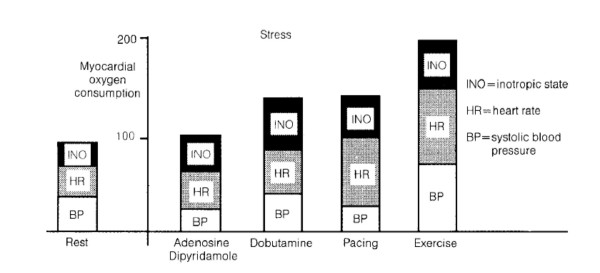
Major determinants of myocardial oxygen consumption in resting conditions (left) and during stress commonly employed with echocardiography.

**Figure 2:**
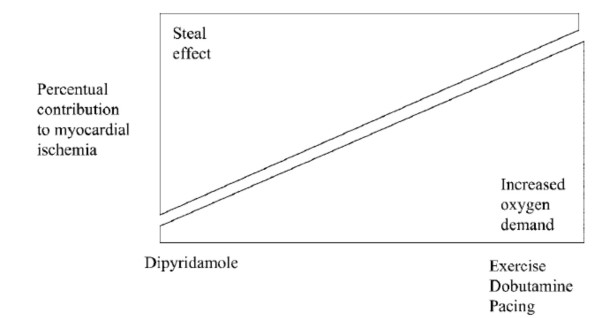
Conceptual allocation of tests employed in combination with echocardiography to detect coronary artery disease stenosis inducing ischemia via steal effect (left) or increased myocardial oxygen demand (right), or both mechanisms.

The answer to all these issues relies on the mechanism through which myocardial ischemia is induced by the different stressors (exercise or pharmacologic such as dipyridamole and dobutamine) employed during stress testing. Test exploring organic coronary artery stenosis can induce ischemia by two basic mechanisms: 1. an increase in oxygen demand, exceeding the fixed supply and 2. flow maldistribution due to inappropriate coronary arteriolar triggered by a metabolic/pharmacologic stimulus [1]. The mechanism of increased demand can be easily fitted into the familiar concept framework of ischemia as a supply-demand mismatch, deriving from an increase in oxygen requirements in the presence of a fixed reduction in coronary flow reserve. The different stresses can determine increases in demand through different mechanisms (Fig. 1). In resting conditions, myocardial oxygen consumption is dependent mainly upon heart rate, inotropic state, and the left ventricular wall stress (which is proportional to the systolic blood pressure) [2]. Following dipyridamole or adenosine administration, a slight increase in myocardial function, a modest decrease in blood pressure, and mild tachycardia can be observed, overall determining only a trivial increase in myocardial oxygen demand [3]. During exercise, the increase in heart rate, blood pressure, and inotropic state accounts for the overall increase in myocardial oxygen consumption [4]. Pacing and dobutamine also increase – to a lesser degree – myocardial oxygen demand [5]. During pacing, the increase is mainly due to the increased heart rate. Dobutamine markedly increases contractility and heart rate. Further augment in myocardial oxygen consumption for heart rate increase occurs with the co-administration of atropine with dobutamine [6]. and dipyridamole [7]. (Fig. 2).

In the presence of coronary atherosclerosis, appropriate arteriolar dilation can paradoxically exert detrimental effects on regional myocardial perfusion, causing overperfusion of myocardial layers or regions already well perfused in resting conditions at the expense of regions or layers with a precarious flow balance in resting conditions [8]. Anti-ischemic therapy can interfere with all the above mentioned mechanisms of ischemia induction, although in a very different fashion. The mechanism of action of anti-ischemic drugs is easily fitted within the familiar framework of supply-demand mismatch. In particular, beta-blockers are credited with reducing exercise-induced ischemia by decreasing myocardial oxygen demand and possibly by increasing supply through a reduction in extravascular forces [9]. Experimental studies demonstrated that beta-blockers reduce dipyridamole-induced ischemia by an alteration of regional myocardial blood flow [10]. However, this straightforward explanation seems inadequate in justifying the protective effects of beta-blockers on dipyridamole-induced ischemia. Experimental data show that beta-blockers do not affect the dipyiridamole-induced increase in flow [12]. On the other hand, the increase in myocardial oxygen consumption does not play any significant role in the induction of dipyridamole-induced ischemia, which is due to an absolute reduction in subendocardial flow (tightly linked to regional wall thickening) mostly for "vertical" and "horizontal" steal phenomena.

However, experimental studies on the model of the exercising dog have shown that beta-blockers protect myocardium from stress-induced myocardial blood flow-function relation: for a given transmural flow, there is a rise of subendocardial and a fall of subepicardial flow, with an improved regional performance [10]. This same mechanism has also been documented with some calcium antagonists, such as diltiazem and may explain, in part, the beneficial effects of this class of drugs on dipyridamole-induced ischemia. Calcium antagonists can effectively prevent ischemia provoked by dipyridamole also through other mechanisms, which they share with nitrates, and they tend to increase the coronary flow supply during stress. In this case, the prevention of steal phenomena may be due to the increase in collateral flow (which has been shown with nitrates and, to a much lesser extent, with some calcium antagonists) [12]. and to the dilation of epicardial coronary lumen size. The pronounced increase in collateral flow can prevent horizontal steal phenomena due to dipyridamole, wheras even a small increase of the coronary diameter can dramatically reduce the blood pressure drop across the stenosis, therefore preventing vertical steal phenomena. Beta-blockers exert a direct and competitive action on beta-1 receptors, as they are employed as specific antagonists of dobutamine-induced ischemia. Dobutamine, through its beta-1 agonist action determines the increase in oxygen consumption, but it induces flow maldistribution through beta-2 arteriolar receptors.

## Diagnostic implications of the use of anti-ischemic therapy during stress testing

**Figure 3:**
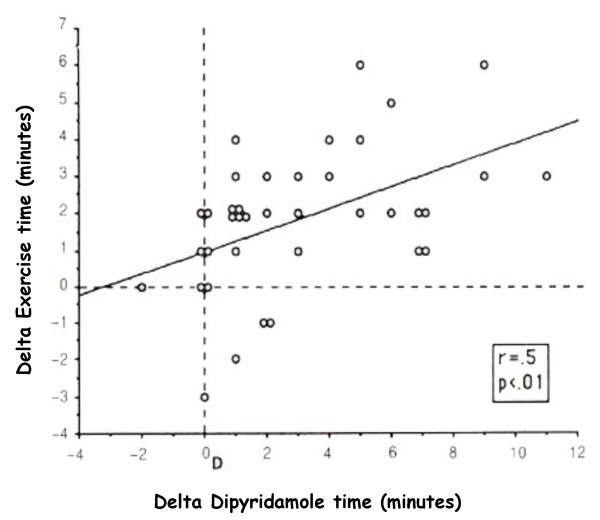
Correlation between the therapy-induced variations in dipyridamole and exercise time in the 38 patients with positivity of both tests off treatment (Modified from 21).

**Figure 4:**
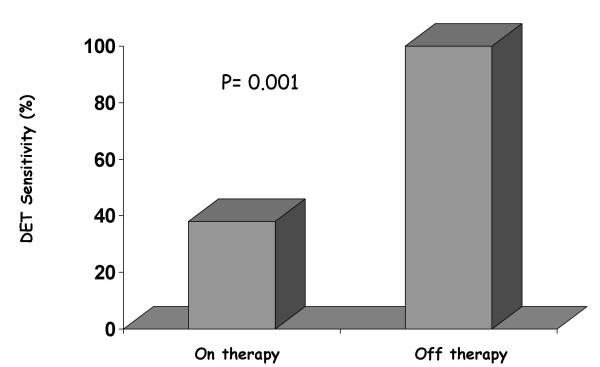
Dipyridamole test sensitivity on and off beta-blocking therapy (Modified from 23). Test sensitivity is significantly reduced in patients studied on beta-blocking therapy.

**Figure 5:**
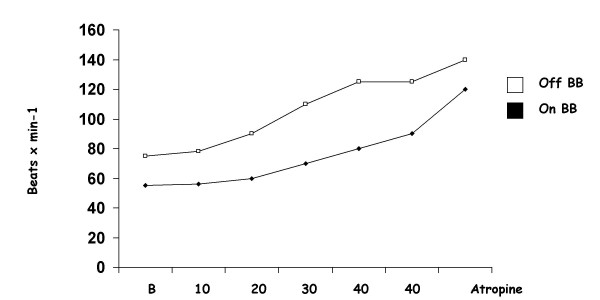
Heart rate during dobutamine-atropine stress testing on and off beta-blockers (Modified from 25).

**Figure 6:**
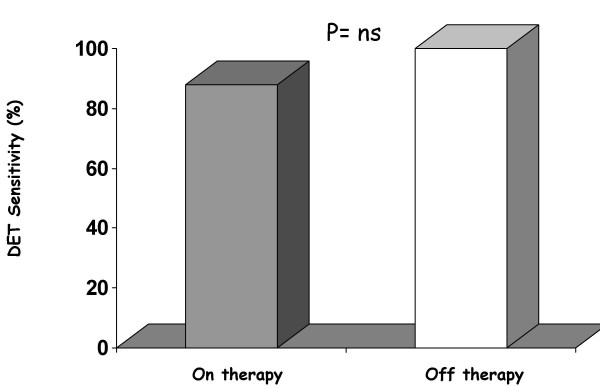
Dobutamine-atropine stress echocardiography test sensitivity in patients on and off non-beta blocking therapy (Modified from 26).

On the basis of these premises and taking into consideration that the markers of inducible myocardial ischemia (electrocardiogram, perfusion, wall motion) are very different therefore expressing a different sensitivity to the action of anti-ischemic therapy at time of testing, it is clear that medical therapy affects test results (see table 1. In fact, the AHA/ACC Guidelines on Chronic stable angina state that [13]. "whenever possible, it is recommended that beta-blockers (and other anti-ischemic drugs) be withheld for four to five half-lives (usually about 48 h) before exercise imaging studies for the diagnosis and initial risk stratification of patients with suspected CAD". Ideally, these drugs should be withdrawn gradually to avoid a withdrawal phenomenon that may precipitate events. When beta-blockers cannot be stopped, stress testing may detect myocardial ischemia less reliably, but it usually will still be positive in patients at the highest risk. The same recommendations apply to imaging stress testing. Nonetheless, in patients who exercise to a submaximal level because of the effect of drugs, perfusion or echocardiographic imaging still affords higher sensitivity than the exercise ECG alone [14]. On the basis of these recommendations patients undergoing a stress testing for diagnostic purposes should be evaluated off therapy not to offset test results. Exercise imaging stress (nuclear perfusion or ultrasound) testing have a lower sensitivity when performed on anti-ischemic therapy [14-19]. due to the limited increase in heart rate and blood pressure which determine oxygen consumption. Antianginal therapy lowers the sensitivity of exercise echocardiography as it does with vasodilator stress testing [19,20]. Antianginal therapy with beta-blockers, calcium-antagonists or nitrates in various combinations prevent dipyridamole-induced ischemia by delaying the appearance of the transient dyssynergy [21]. this variation on dipyridamole time parallels variations in exercise time at exercise stress testing [21]. (Fig. 3). Dipyridamole stress sensitivity was 91% off therapy and fell to 65% under therapy in various combinations (beta-blockers and/or calcium antagonists and/or nitrates). The same reduction of dipyridamole test sensitivity is obtained with monotherapy with beta-blockers at time of testing (100% off therapy vs. 38% on therapy) [22,23]. (Fig. 4). Interestingly, the positive effects of beta-blockers on dipyridamole stress are largely independent of the effect on heart rate, possibly involving a direct anti-steal effect [21-23]. Angiotensin-converting enzyme inhibitors have no effect on dipyridamole stress echocardiography results [24]. The sensitivity of dobutamine is heavily affected by concomitant beta-blocker therapy. Beta-blockers effect a rightward shift in the dose-response curve to dobutamine and sharply lower test sensitivity, unless atropine is used [25]. (Fig. 5). Calcium antagonists and/or nitrates only mildly reduce dobutamine stress sensitivity (100% off therapy vs.88% on therapy, p=ns) (Fig. 6). Non-beta-blocker antianginal therapy reduces the severity of dobutamine-induced ischemia by reducing the value of peak wall motion score index and time of ischemia appearance. However, these changes are not correlated to variations in exercise tolerance [26].

**Figure 7:**
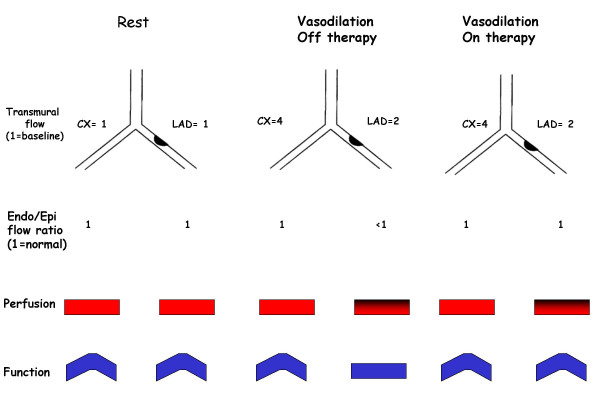
Schematic illustration of the principle underlying the impact of antianginal therapy on different markers of ischemia: regional function and perfusion imaging. At rest, perfusion is homogeneously distributed between endocardial and epicardial layers. In the presence of a significant coronary stenosis, vasodilation induced by pharmacologic stress, provokes a subendocardial underperfusion with a relative epicardial overperfusion which is translated into an impairment of function (echocardiographic dyssynergy) and perfusion (reversible defect at scintigraphy). Medical therapy at time of testing re-equilibrates the imbalance between subendocardial and subepicardial layers, but it affects only function.

Dipyridamole stress nuclear imaging techniques do not seem to be influenced by anti-ischemic therapy [27]. However, it has been recently demonstrated that acute administration of beta-blockers in patients with known coronary artery disease reduces dipyridamole SPECT sensitivity from 69% with placebo to 52% (p = 0.039) with 10 or 20 mg of metoprolol in a per-vessel analysis, but not overall sensitivity [28]. The reason for this difference between stress echocardiography and nuclear imaging is likely to be related to the different markers of ischemia, i.e. wall motion abnormalities vs. perfusion, in the face of the same pathophysiologic mechanism of ischemia: the reduction of coronary reserve. In fact, in the presence of a coronary stenosis, during stress, flow remains elevated in the subepicardial layer but falls in the subepicardium. This selective stress-induced hypoperfusion is important for stress echocardiography, since the regional systolic thickening is linearly and closely related to subendocardial perfusion and only loosely related to subepicardial perfusion [29,30]. (Fig. 7).

**Figure 8:**
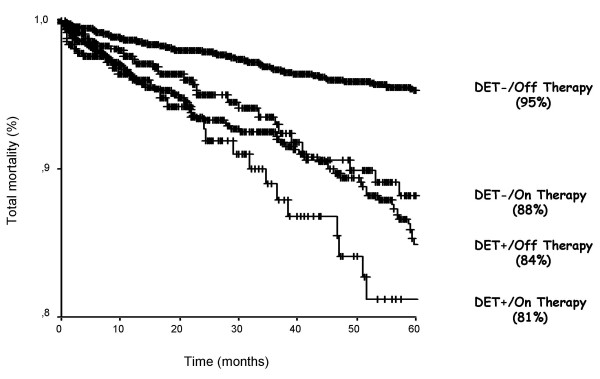
Kaplan-Meier survival curves (considering total mortality as an endpoint) in patients stratified according to presence (DET +) or absence (DET -) of myocardial ischemia at pharmacological stress echocardiography on and off antianginal medical therapy. The best survival is observed in patients with no inducible ischemia off therapy; the worst survival in patients with inducible ischemia on therapy (Positive DET vs. Negative DET off antianginal medical therapy, p < 0.000; Positive DET vs. Negative DET on antianginal medical therapy, p < 0.074) (Modified from 31).

## Prognostic implications of anti-ischemic therapy during stress testing

The protective effect of anti-ischemic therapy on inducible myocardial ischemia might exert a powerful impact on prognosis. From the EPIC-EDIC Data bank, it has been analyzed the prognostic impact of antianginal therapy at time of testing in 7333 patients with suspected or known coronary artery disease undergoing pharmacologic stress echocardiography with either dipyridamole or dobutamine. The results show that a positive test on medical therapy is an additional marker of ischemia severity at stress testing whereas a negative test on medical therapy is less prognostically benign, being a false negative result [31]. (Fig. 8). No prognostic difference was found among the various forms of anti-ischemic drugs at time of testing, but the presence per se of antinaginal therapy at time of testing is an independent predictor of death. It is worth noting that in the study only a very low percentage of patients was taking beta-blockers: if on one side this aspect represent a clear lack of adherence to recommendations [13]., on the other it is an observed pattern of prescription in our data base, which simply reflected the clinical practice and the lack of a universally accepted policy of testing regarding concomitant therapy [32,33]. Marwick et al. [34]. have demonstrated a protective effect on mortality of beta-blocker therapy in patients with a negative exercise echocardiography whereas specificity and negative predictive value is increased for the prediction of cardiac events (cardiac death, myocardial infarction and unstable angina) during exercise scintigraphy in patients evaluated off medical therapy at time of testing [35].

The clinical implications of these results are far-reaching. Inducible myocardial ischemia during pharmacological stress testing on medical therapy identifies the subset of patients at highest risk of death. On these patients an aggressive approach has to be undertaken in order to change the natural history of coronary artery disease. On the far opposite end the incidence of death in patients with a negative pharmacologic test off therapy is so low that no intervention could lower the spontaneous rate of death any further. At intermediate risk are those patients with a negative test on medical therapy or with a positive test off medical therapy. Different clinical scenarios can be foreseen on the basis of the present results: 1) A negative test on medical therapy might represent a false negative result, therefore it is advisable to repeat the test off therapy in order to assess the real ischemic burden through the conventional stress echocardiographic parameters [36,37]. – i.e. number of ischemic segments, severity of induced dysfunction, (both expressed by peak wall motion score index), pharmacologic load and time of onset of ischemia. This is in line with the recommendations of the American Heart Association in patients with stable angina [13].; 2) In the case of a positive test off medical therapy, the effect of therapy can be assessed with the advantage of using an objective, primary ischemic end point such as changes in wall motion during stress.

## Conclusions

Patients may be undergoing various forms of antianginal therapy at the time of testing, both an advantage and a disadvantage for stress echocardiography testing. The disadvantage is that antianginal therapy reduces sensitivity, since stress-induced wall motion abnormalities are caused by the development of obligatory myocardial ischemia. The advantage is that the effect of therapy can be assessed using an objective, primary ischemic end-point such as changes in stress-induced wall motion abnormalities. The presence of ischemia can be titrated on the basis of the ischemic-free stress time and the extent and severity of the induced dyssynergy. The various forms of stress are differently affected by various forms of therapy. In patients with known or suspected coronary artery disease, ongoing anti-ischemic therapy at the time of testing heavily modulates the prognostic value of pharmacological stress echo. In presence of concomitant anti-ischemic therapy, a positive test is more prognostically malignant and a negative test less prognostically benign. However, the decision to remove a patient from beta-blocker therapy for stress testing should be made on an individual basis and should be done carefully to avoid a potential hemodynamic rebound effect, which can lead to accelerated angina or hypertension [38]. No major side effects were recognized when medical therapy was withdrawn in large series of consecutive patients undergoing pharmacologic stress echocardiography [39]. In practical terms, when a test is performed for diagnostic purposes it should be done off medical therapy in order to avoid the influence of medical therapy (in case of hypertensive patients it is possible to prescribe ACE-inhibitors or Angiotensin II receptor blockers that do not exert any protective effect on myocardial ischemia). In patients with known coronary artery disease the decision to suspend medical therapy should be taken on an individual basis in view also of the fact that pharmacologic stress echocardiography is a versatile tool that can assess medical therapy efficacy in the long term prognosis [31].

**Table 1:** Effects of oral therapy on stress testing sensitivity

	**STRESS**		
	**Exercise**	**Dipyridamole**	**Dobutamine**
Beta-blockers	↓	↓	↓↓
Calcium channel blockers	↓	↓	↓↔
Nitrates	↓	↓	↓↔
ACE-inhibitors	↔	↔	↔
Aminophylline	↓↔	↓↓	↔

ACE, angiotensin-converting enzyme; ↓, decreased sensitivity; ↓↓ markedly decreased sensitivity; ↔, no effect on sensitivity; ↓↔, mild decrease in sensitivity.
